# Biomedical Applications of Deformable Hydrogel Microrobots

**DOI:** 10.3390/mi14101824

**Published:** 2023-09-24

**Authors:** Qinghua Cao, Wenjun Chen, Ying Zhong, Xing Ma, Bo Wang

**Affiliations:** 1 School of Materials Engineering, Shanghai University of Engineering Science, Shanghai 201620, China; cqh971201@163.com; 2 School of Materials Science and Engineering, Harbin Institute of Technology (Shenzhen), Shenzhen 518055, China; zhongy@hit.edu.cn (Y.Z.); maxing@hit.edu.cn (X.M.); 3 Sauvage Laboratory for Smart Materials, Harbin Institute of Technology (Shenzhen), Shenzhen 518055, China

**Keywords:** hydrogel robots, biological applications, shape deformation, stimuli-responsive

## Abstract

Hydrogel, a material with outstanding biocompatibility and shape deformation ability, has recently become a hot topic for researchers studying innovative functional materials due to the growth of new biomedicine. Due to their stimulus responsiveness to external environments, hydrogels have progressively evolved into “smart” responsive (such as to pH, light, electricity, magnetism, temperature, and humidity) materials in recent years. The physical and chemical properties of hydrogels have been used to construct hydrogel micro-nano robots which have demonstrated significant promise for biomedical applications. The different responsive deformation mechanisms in hydrogels are initially discussed in this study; after which, a number of preparation techniques and a variety of structural designs are introduced. This study also highlights the most recent developments in hydrogel micro-nano robots’ biological applications, such as drug delivery, stem cell treatment, and cargo manipulation. On the basis of the hydrogel micro-nano robots’ current state of development, current difficulties and potential future growth paths are identified.

## 1. Introduction

Robots have developed expeditiously in the macroscopic world in recent decades, including industrial robots, service robots, underwater robots, and space robots. Meanwhile, in the micro/nanoscale world, micro/nanorobots have also received tremendous attention owing to their autonomous motion. Micro/nanorobots are untethered devices capable of navigating into small spaces and conducting controlled missions. They are ideal for a number of biomedical applications, such as targeted drug delivery [1], microsurgery [2], biosensing [3], and biological sampling [4]. Most of the microrobots created to date are composed of rigid materials, which makes it difficult for them to handle fragile objects. Moreover, the microrobots can hardly bend or deform, which restricts their ability to advance in biomedical applications [5]. Low-modulus and biocompatible hydrogel materials are the ideal multipurpose materials to construct shape-changeable soft microrobots in biomedical fields, especially when performing the delicate manipulation of bioentities in challenging environments, because of their high biocompatibility and conformation-adaptability.

Hydrogels are composed of a crosslinked water-swollen network which is similar to the structure of biological tissues, making them exhibit excellent biocompatibility, softness, and ability to imitate living organisms. One of the ultimate goals for intelligent micro/nanorobots’ development is to mimic micro creatures in nature so that they can adapt to a complex environment. Thus, smart hydrogel, which can respond to external stimuli, is a suitable candidate to develop microrobots or actuators. Usually, stimuli-responsive hydrogels can deform upon exposure to environmental stimuli, such as pH, temperature, light, magnetic field, electric field, humidity, and so forth.

**Scheme 1:**
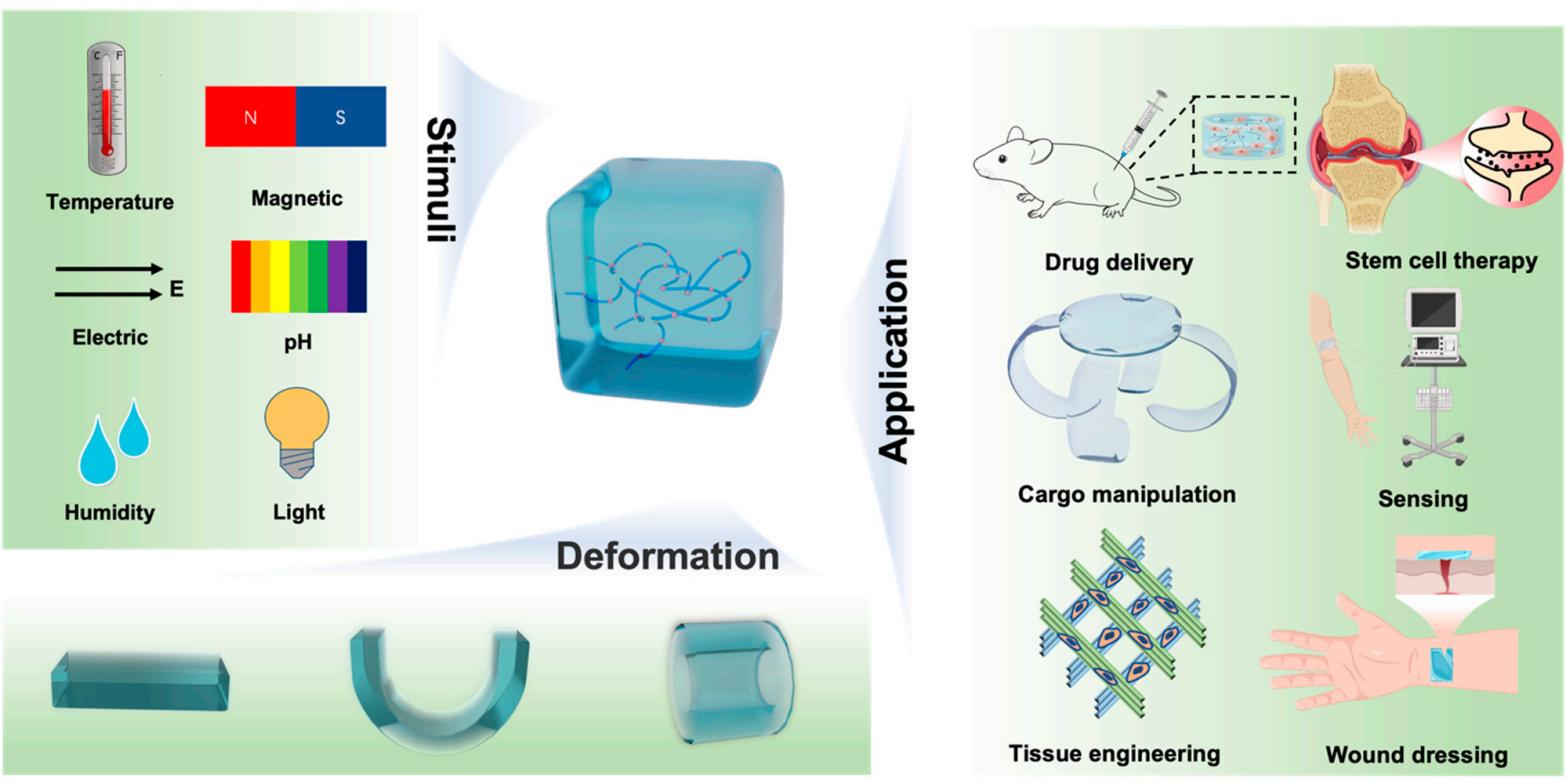
Stimuli-responsive hydrogels and intelligent microrobots/actuators.

The intelligent microrobots made of hydrogel are used for many biological applications, such as drug/cell delivery [6], wound healing [7], implantable devices [8], etc., as shown in Scheme 1. However, there are still several challenges that need to be overcome before further development can be achieved in the use of intelligent microrobots for biomedical or bioengineering applications. For example, the biocompatibility and biodegradability of the hydrogel, the integration of multiple functions in micro- to nano-scale systems, the biocompatibility of the external stimulus, the limited mechanical modes of the intelligent microrobots, etc.

This mini-review article will introduce the stimulus-responsive deformation mechanisms of the smart microrobots/actuators, including the hydrogel materials, the structural designs, and the fabrication methods. Then, we will go over the representative deformable-hydrogel-based microrobot in biomedical applications, as well as the challenges and potential for smart microsystems.

## 2. Mechanism

When it comes to smart microrobots and actuators, hydrogel, which can respond to various environmental stimuli, plays an important role. The deformation of hydrogels typically results from inhomogeneous stimulus-induced responses forming a difference between the responding area and the non-responsive area to generate the deformation [9]. These hydrogels have specific responses to physical or chemical signals. The mechanisms of the deformation can be categorized by the various stimuli, including light, pH, temperature, electric and magnetic fields, etc., which are outlined and discussed in this section [10]. We also summarized the most commonly used hydrogel materials, the preparation method, and the applications in Table 1.

Hydrogel formation involves several processes within an aqueous solution, primarily relying on two distinct crosslinking mechanisms: physical crosslinking and chemical crosslinking. Physical crosslinking, while simplifying gelation without altering the polymer chain, lacks precision in controlling hydrogel properties. Nevertheless, it boasts reversibility and eliminates the need for toxic organic solvents and small molecule crosslinking agents, rendering hydrogels biocompatible and biodegradable. However, due to the weaker intermolecular interactions compared to chemical bonds, physical crosslinking often results in hydrogels with suboptimal mechanical strength and stability. Conversely, chemical methods offer a more precise and controlled approach to the crosslinking process. In Table 2, we provide a summary of the most widely employed crosslinking processes.

### 2.1. Temperature

**Figure 1:**
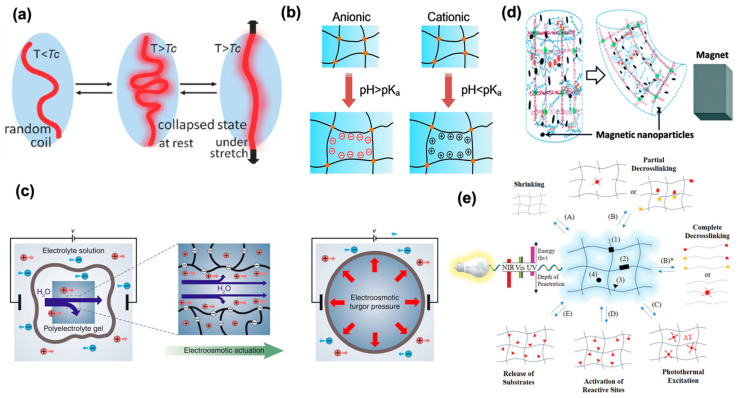
(**a**) Temperature-response hydrogel by the LCST phase-transition of the PNIPAm network. Reproduced with permission [44]. Copyright 2016, Wiley-VCH. (**b**) Schematic of the actuation mechanism of the pH-responsive hydrogels. Reproduced with permission [41]. Copyright 2019, Springer Nature. (**c**) Schematic of the electroosmotic actuation process of a hydrogel expansion actuator in an electrolyte solution. Reproduced with permission [45]. Copyright 2022. The Authors, some rights reserved, exclusive licensee American Association for the Advancement of Science. (**d**) Schematic of magnetically induced hydrogel bending. Reproduced with permission [46]. Copyright 2015, Royal Society of Chemistry. (**e**) Molecular structure and response of photosensitive hydrogels. Location of photoresponsivity groups: (1) in the crosslinking points, (2) along the polymer or supramolecular backbone, (3) along the side chains, (4) dissolved in the aqueous medium of hydrogels. Photoresponses type: (A) shrinking, (B) partial de-crosslinking, (C) photothermal excitation, (D) activation or deactivation of reactive sites, (E) release or capture of substrates. Reproduced with permission [47]. Copyright 2019, Wiley-VCH.

One of the external stimulus factors is temperature, which is the most widely used inspiration for materials morphing because of its easy controllability. The critical solution temperature is an important index for hydrogel since the primary response mechanism of thermosensitive hydrogel is its solubility change in aqueous solution as a function of temperature [41]. A lower critical solution temperature (LCST) is defined as the temperature above which the material has a phase transition from a soluble status to an insoluble status. Correspondingly, the upper critical solution temperature (UCST) is the temperature when the phase transition happens in the opposite way [42]. One of the most representative materials is poly N-isopropylacrylamide (PNIPAM), a well-characterized poly (N-alkyl substituted acrylamide) polymer. It shows a change from hydrophilicity to hydrophobicity when the temperature goes above the LCST point. As shown in Figure 1a, when the temperature falls below the LCST, PNIPAM exhibits a water-swollen, loose hydrogel network, showing hydrophilicity. By contrast, the chain shows a collapsed and compressed conformation when the temperature goes above the LCST. As a result, the hydrogel network releases water, leading to a shrunken state on the macroscopic scale. Moreover, the LCST of PNIPAM is around 32 °C [43], which makes it popular for biomedical applications.

**Figure 2:**
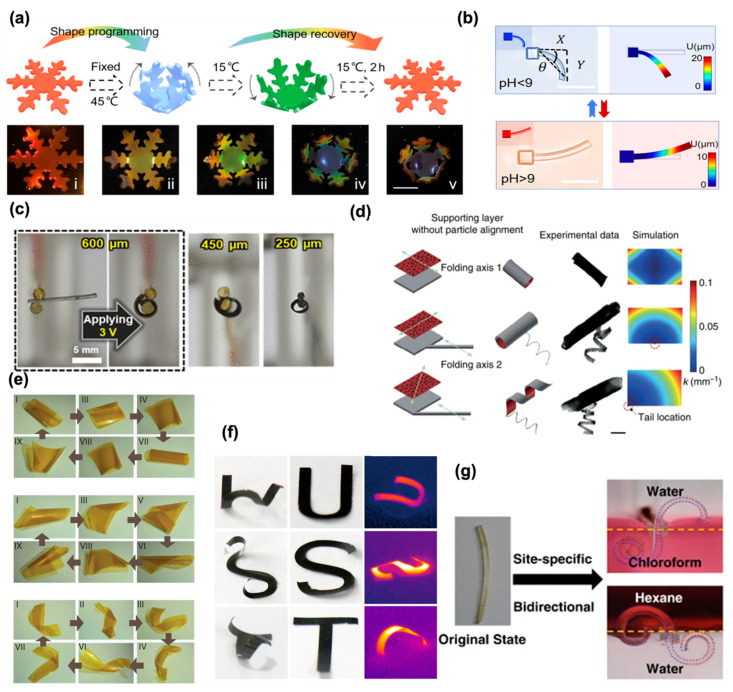
(**a**) Schematics for the programmable process of the snowflake shaped SMSC hydrogel film. Optical images of the snowflake shaped SMSC hydrogel film in 45 °C water. The bending angles in (**b**): (i) 0°, (ii) 30°, (iii) 50°, (iv) 80°, (v) 90°. Reproduced with permission [11]. Copyright 2022, ELSEVIER. (**b**) Microcantilever bending and recovery are induced by changes in pH values, where θ is the bending angle. Reproduced with permission [15]. Copyright 2021, American Chemical Society. (**c**) Photographic images of WNE actuators with different thicknesses operating at 3 V. Reproduced with permission [19]. Copyright 2022. The Authors, some rights reserved, exclusive licensee American Association for the Advancement of Science. (**d**) Formation of compound micromachines with a bilayer head and monolayer tail. Due to the lack of particle alignment, the folding axis of the head is solely determined by the edge effect. Finite element modeling (FEM) simulations visualize internal stress distribution. Reproduced with permission [22]. Copyright 2016, Springer Nature. (**e**) Mechanism of the hygroinduced locomotion of PCAD@AG films. For each aspect ratio and stage of locomotion, both cartoons and snapshots of the real film are shown. Top I–IX: Square-shaped film, middle I–IX: rectangular film and bottom I–VIII: film strip. Reproduced with permission [26]. Copyright 2015, Springer Nature. (**f**) the structural changes of actuators with H-, U-, S-, and T-shape patterns under the stimuli of humidity and IR light. Reproduced with permission [25]. Copyright 2019, Springer Nature. (**g**) Site-specific bending of the Janus film. Reproduced with permission [48]. Copyright 2017, Springer Nature.

Wang et al. prepared SMSC hydrogel films with an inverse opal scaffold structure on shape memory polymers (SMPs) based on N-isopropylacrylamide (NIPAM) and stearyl acrylate (SA) copolymers by the sacrificial template method [11]. By introducing photothermal responsive graphene quantum dots, the hydrogel film could be endowed with photo-controlled reversible deformation and structure color change behavior. The hydrogel film showed a bending state at 45 °C, and when it was placed in water at 15 °C, it was able to be restored to its original shape, as shown in Figure 2a. In multiple cycle tests, the SMSC hydrogel film showed good reversible deformation ability. Furthermore, due to its advantages in shape variation, specific shapes have been designed for use in biomedicine. The claw-shaped hydrogel gripper can grasp and release a glass ball by tuning the temperature, thus accomplishing cargo transport. Therefore, based on temperature-responsive hydrogels, the deformation motion of microrobots or actuators can be realized by adjusting the temperature. More details about the representative research work can be found in Table 1.

### 2.2. pH

pH-responsive hydrogels are sensitive to the pH changes in the surrounding environment, and correspondingly exhibit deformation behaviors of expansion and contraction. Generally speaking, pH-sensitive deformation is related to the functional groups on the polymer chain. When the pH value of the surrounding environment changes, these groups are ionized, which leads to the dissociation of the hydrogen bonds between the molecular chains, resulting in electrostatic interaction. Such interaction causes either the absorption or release of the water in the hydrogel network, leading the hydrogel to swell or shrink. As shown in Figure 1b, according to the classification of group properties, pH-responsive hydrogels can be divided into anionic hydrogels, cationic hydrogels, zwitterionic hydrogels, and so on.

Polyacrylic acid (PAAC) is a typical pH-responsive hydrogel material. The functional group on the PAAC chain is carboxyl, which is easily protonated at a low pH. On this basis, Chen et al. fabricated deformable hydrogel microrobots (SMMRs) based on polyacrylic acid (PAAC) using a one-step 4D laser printing technique [15]. At pH > 9, the deprotonation of the carboxyl group is negatively charged, which generates electrostatic repulsion between the molecular chains, resulting in the swelling of the hydrogel. On the contrary, at pH < 9, the hydrogel shrinks (Figure 2b). Based on the reproducible shape switching of the SMMRs to open and close claws via pH-responsiveness, crab-shaped SMMR hydrogel micro-actuators are able to pick up and release 10 μm silica particles. For other pH-responsive hydrogels based on polyacrylamide (PAAM), researchers took advantage of the anisotropic mechanical properties of hydrogel actuators (SNPP) with silver nanoparticles/polyacrylamide (PAAM) [18]. By employing metal modules that have been treated with thioic acid as multipurpose crosslinking agents, an anisotropic hydrogel made of highly ordered noble metal nanostructured module sheets was created. Under near-infrared and low pH conditions, the SNPP hydrogel exhibited a self-healing behavior. pH-responsive hydrogels are also the main branch in the hydrogel system so there are more prospects that need to be studied and discovered.

### 2.3. Electric Field

Electro-responsive hydrogels can expand/shrink under the support of an electric field. The mechanism of the deformation is mainly considered to be related to the directional movement of free ions in solution under an electric field. In the hydrogel–electrolyte system, free ions migrate directionally under the action of an electric field, resulting in uneven ion concentrations inside and outside the hydrogel [45]. Thus, the electroosmosis can directly generate a dragging force on the fluids inside the hydrogel network and the osmotic pressure can cause hydrogel deformation (Figure 1c).

Based on the conversion of electrical energy to mechanical energy, there has been plenty of research on related hydrogel systems taking advantage of electro-responsiveness. Chitosan, PAAC, 4-hydroxybutyl ester (4-HBA), and poly (2-acrylamido-2-methylpropanesulfonic acid) (PAMPS) are all candidates for electro-responsive hydrogel materials [41]. Among them, PAAC is a popular material; the carboxyl groups on the polymer chain are easily dissociated to allow the hydrogel to obtain ion conductivity. As we mentioned previously, PAAC-based hydrogels are also pH-responsive. Cho et al. combined a gold nanoparticle layer with a polyacrylic acid-polyacrylamide (PAA-co-PAAm) hydrogel to fabricate wrinkled nanomembrane electrode (WNE) hydrogel actuators [19]. The formation of these solid metallic NP layers on the hydrogel surface was caused by the vertical and horizontal growth of densely arranged metallic nanoparticle (NP) arrays on the wet hydrogel surface, which was induced by the continuous capillary transport and ligand exchange reaction of amine-functionalized polymer linkers in an aqueous gel in non-polar media. They realized a low-voltage drive that can show the bending deformation of the hydrogel under a voltage of 3 V, as shown in Figure 2c. At the same time, the WNE hydrogel driver also had excellent mechanical deformation capacity (strain exceeds 50%) and high electrical conductivity. Compared with stimuli like temperature, pH, and light, the electro-responsive deformation can overcome the intrinsic diffusion-limited water transport. In other words, electrically triggered osmosis is a relatively more efficient deformation mode.

### 2.4. Magnetic Field

Different from the above-mentioned deformation mechanisms, a response to a magnetic field is not an inherent property of the hydrogel itself, but the hydrogel is modified by additives to achieve magnetic responsiveness, as shown in Figure 1d. So far, Fe_3_O_4_ nanoparticles, Co nanoparticles, NdFeB nanoparticles, and so on, have been introduced as magnetic additives into hydrogel robots or actuators. Among them, Fe_3_O_4_ nanoparticles are the most widely used due to their superparamagnetism, biocompatibility, and non-cytotoxicity.

As long as the magnetic particles are mixed in hydrogels, magnetic responsiveness can be imparted, so there are a variety of hydrogel matrices that can become magnetically responsive hydrogels. Huang et al. [22] patterned a thermoresponsive N-isopropylacrylamide (NIPAM) hydrogel layer on a non-swellable supporting hydrogel layer composed of polyethylene glycol diacrylate (PEGDA) wherein the bilayer hydrogel structure undergoes self-folding deformation by aligning the axes of magnetic Fe_3_O_4_ nanoparticles (Figure 2d). Then, under the action of a magnetic field, the helical hydrogel driver can be rotated and propelled. The combination of a hydrogel and magnetic particles enables the hydrogel to respond and drive in a tunable and wireless manner according to the guidance of the magnetic field, which is a great advantage for further research and development of magnetically responsive hydrogels.

### 2.5. Light

Light is a stimulus with advantages such as low cost, remote manipulation, biocompatibility, and controllability. Similar to other stimuli-responsive mechanisms, the deformation of the hydrogel comes from water absorption or the release of the networks. The water uptake-induced swelling is mainly determined by hydrophilicity and the crosslinking density. A photoisomerization reaction can influence the crosslinking of hydrogel and the light-induced cleavage of polymer backbones or crosslinking points also can modulate the crosslinking, which will affect the hydrogel’s swelling property [47]. The mechanism is shown in Figure 1e. Furthermore, the light-induced deformation can be combined with other stimuli. For example, hydrogels’ morphing can be achieved by utilizing photothermal or photochemical reactions. The heat generation by light irradiation can lead to reversible phase transitions of the temperature-responsive polymers. Zhang et al. used hydrogels doped with photoactive dopants to realize the shrinkage behavior of hydrogels under the control of weak ultraviolet light [26]. An intelligent hydrogel material was created by fusing the robust moisture absorption capabilities of a natural hydrogel (AG) with the photoactivity of a flexible synthetic photoactive polymer based on a photoactive polyethylene glycol coupling compound that contains azobenzene. Both the moisture gradient and the photomechanical reaction sparked by ultraviolet light caused the material to react. The hydrogel driver has two driving modes and can move between them. Zhu and his colleagues used coumarin as a photo-crosslinking unit to form patterned gradient structures in a hydrogel, guiding the deformation to various configurations and demonstrating the hydrogel’s programmability [49]. They created hydrogels using micellar polymerization with hydrophobic Coumarin monomer and hydrophilic acrylic acid as the starting components. Coumarin efficiently photo-dimerized and photo-dissociated under the illumination of 365 nm and 254 nm, respectively, and accomplished reversible changes in the structure of the hydrogel network. Different gradient structures gradually create patterns in a hydrogel using photolithography, creating various shapes.

### 2.6. Others

In addition to the aforementioned stimulus-responsive mechanisms, there are also other stimulation signals, such as humidity [50], biomolecules [51], redox [52], and solvents [53]. For example, the hydrogel actuator prepared by Zhang and co-workers [26] utilized the humidity gradient to realize non-uniform swelling, where water absorption induced significant deformation. (Figure 2e). The hydrogel actuator is always twisted or curled away from the source of moisture because the exposed side expands as a result of water absorption on that side. The hydrogel actuator swiftly twists and unfolds when placed over wet filter paper, moving in turn in the opposite direction and moving quickly on the paper. At the same time, the hydrogel actuator can transport a cargo of nearly 20 times its own weight. Inspired by natural organisms, researchers have designed a double-layer driver that can bend at a high speed and amplitude according to changes in environmental humidity [54]. The hydrogel actuator produced unusually high bending angles and speeds under near-infrared (NIR) light or humidity when normalized to variations in thickness and temperature. The asymmetric structure design of directional control jumping, which was inspired by frog hopping, makes it possible. For solvent responsiveness, a bidirectional bending of hydrogels in an acetone–water mixed solution system was achieved, as well as specific bending in a water/organic solvent system [48], shown in Figure 2g. The binary hydrogen/organic copolymer membranes that make up the deformable Janus membrane were successfully synthesized in one step by layering an immiscible monomer solution. Bidirectional bending was achieved in the acetone–water mixed solution system using the special phase transition properties of the PAA network, and particular site bending in the water/organic solvent system.

## 3. Fabrication and Shape-Morphing Design

As discussed previously, hydrogels usually undergo expansion or contraction by absorbing or releasing water upon external stimulation. However, a simple swell-up will not meet the functional requirement of the microrobots/actuators. Thus, in order to achieve intelligent shape-morphing for accomplishing functions, the structure needs to be designed and fabricated deliberately. In this chapter, we will introduce the common fabrication methods and the design of structural deformation.

### 3.1. Fabrication Methods

#### 3.1.1. Mold Casting

**Figure 3:**
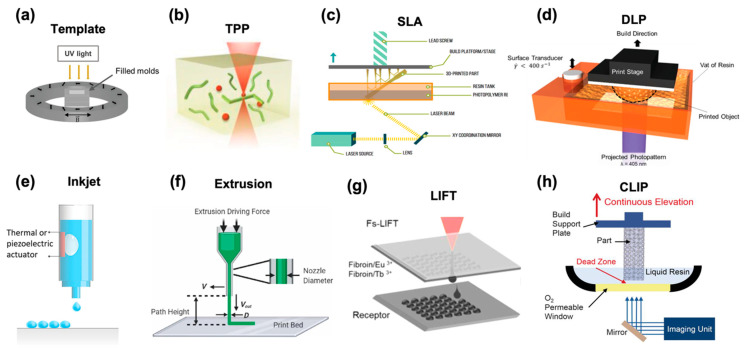
(**a**) A homogeneous magnetic field induced by a Halbach array of 9 mT is applied after the perfluorinated molds are filled. This allows the formation of maghemite dipolar chains. The fixation of the dipolar chains is achieved by the UV-crosslinking of the polymeric network. Reproduced with permission [66]. Copyright 2023, Wiley-VCH. (**b**) Schematic representation of two-photon polymerization by a femtosecond laser inside a DMOF crystal. Reproduced with permission [67]. Copyright 2022, Wiley-VCH. (**c**) Stereolithography process. Reproduced with permission [68]. Copyright 2017, American Chemical Society. (**d**) Schematic of AL-DLP 3D printing hardware. Reproduced with permission [69]. Copyright 2021, Wiley-VCH. (**e**) Inkjet printing. Reproduced with permission [59]. Copyright 2020, American Chemical Society. (**f**) Major process parameters in extrusion bioprinting. Reproduced with permission [70]. Copyright 2021, IOP Publishing Ltd. (**g**) Laser-induced forward transfer. Reproduced with permission [71]. Copyright 2020, American Chemical Society. (**h**) Schematic of CLIP printer. Reproduced with permission [64]. Copyright 2015. The Authors, some rights reserved, exclusive licensee American Association for the Advancement of Science.

Mold casting is a common method for preparing hydrogels. The mold is designed and prepared in advance, and then the corresponding treatment is carried out according to the different reaction conditions of hydrogel formation (physical/chemical crosslinking, photopolymerization) until the hydrogel is crosslinked (Figure 3a). It is a relatively simple and low-cost method that is convenient for preparing various shapes. At the same time, mold casting can be used modularly to prepare hydrogel robots. Zheng and his colleagues used photoresist to pre-make molds with the help of masks, and then modularly prepared hydrogel robots by electrodeposition [55]. However, this technique is only suitable for open wound areas, such as skin-surface wound healing and cardiac hemostasis recovery and can only be employed for large-scale hydrogel models on a macro scale. It is no longer appropriate for making hydrogels with better precision. In the future, there are significant prospects for large-scale and mass-production applications; however, when it comes to complex graphic structures, there still exist substantial challenges that need to be addressed.

#### 3.1.2. Photopolymerization

With the development of micro/nanomanufacturing technology, the fabrication method of hydrogel-based microrobots has also kept pace with the times, i.e., 3D printing has been applied. Two-photon polymerization (TPP) is one of the most important 3D printing technologies for hydrogel microrobots with ultra-high resolution (100 nm) [56]. Due to the nature of nonlinear excitation, the photopolymerization of monomer and photo initiator molecules is caused at the focus of the NIR laser beam without affecting other regions, as shown in Figure 3b. The geometry printed by two-photon polymerization can be easily customized by changing the focusing trajectory of the laser beam. Therefore, TPP has great potential in the precision manufacturing of microrobots with customized and complex structures that can be used for promising applications such as drug/cell/protein/gene delivery. However, the facility of two-photon polymerization is complicated and expensive; therefore, other 3D printing methods have also attracted great attention in the fabrication of smart microrobots. Stereolithography (SLA) is the most common resin 3D printing process (Figure 3c), it generally includes a solution tank, an ultraviolet laser source, a system that allows for the horizontal movement of the laser beam, and a system that controls the vertical movement of the manufacturing platform [57]. Its accuracy is not as high as TPP, but it can be easily combined with other manufacturing technologies to realize preparation, such as mold template assistance function. It plays a significant part in the production of micron-level hydrogel robots and has the ability to directly produce batches of three-dimensional hydrogel robots. The principle of digital light processing (DLP) is similar to that of SLA, but the light source is selectively obscured during the process, i.e., each layer is fully exposed, rather than a point-by-point exposure (Figure 3d) [58]. The advancement of hydrogel robots is significantly aided by these technologies. In addition, a variety of functional nanoparticles have been incorporated into hydrogel formulations to generate integrated multi-functional microrobots for intended applications. However, the prepared liquid must meet a lot of standards for these technologies. This method has a unique hurdle in that maintaining the light source necessary to start the polymerization reaction requires the transmittance of the prepared liquid or the absorbance of the dopant. This technology plays a crucial role in creating fine microstructures and offers a reliable manufacturing process for the use of micro- and nanorobots both within and outside the human body, as well as in biomedicine. However, the stringent standards for production equipment and output limit further progress, demanding further investigation and exploration.

#### 3.1.3. Inkjet

Similar to the manufacturing of other polymers, inkjet is also widely used in stimuli-responsive hydrogel. The working principle of inkjet is to obtain the digital data of the designed model on the computer and form a preset object on the substrate through ink droplets. The most commonly used droplet generation mechanisms include piezoelectric and thermal induction [59,60], shown in Figure 3e. The advantages of inkjet technology include low cost, high printing speed, and non-contact printing to avoid pollution. Compared with other 3D printing methods, inkjet printing has a relatively low resolution (50 mm), which may limit the accuracy of manufacturing microrobots using inkjet printing methods. In the future, this technology holds the potential for rapid and cost-effective manufacturing in the field of biomedicine. However, its current limitation lies in its relatively low manufacturing accuracy, limiting its applicability to large-size structures. This constraint hinders the full realization of the technology’s potential and underscores the need for ongoing research and development efforts to address this challenge.

#### 3.1.4. Extrusion

Micro-extrusion technology (Figure 3f) is the process of loading biomaterials into a barrel and using a pneumatic or mechanical force to squeeze the materials into a predetermined position on the manufacturing platform or stage through a nozzle, and then depositing the final structure layer by layer with a computer-controlled movable printing nozzle [61]. Micro-extrusion printing is suitable for manufacturing relatively large-sized soft robots, and it is able to squeeze biological materials with high cell density. The hydrogel robot’s failure to match design specifications, despite having higher production accuracy and a lower scale, is a drawback. This is very important for printing hydrogel microrobots with good biocompatibility and has important applications in drug/cell delivery. Nevertheless, this technique enhances the production of biomaterials, offering high precision that is particularly advantageous for large-scale bio-robot manufacturing. It opens up a wide range of biomedical applications, including drug delivery. However, producing miniature structures using this technology presents notable challenges and limitations.

#### 3.1.5. 4D Printing

The essence of 4D printing is to add a time dimension to 3D printing. The deformable material will become the desired shape within a certain time. Based on 3D printing, 4D printing incorporates intelligent materials that can react to external environmental changes, such as changes in temperature, humidity, pH, magnetic fields, electric fields, etc. It also employs specific printing techniques to dynamically modify the internal and geometrical attributes of the printed object in reaction to these external inputs. This capability enables the creation of structures that can adapt, transform, or self-assemble in response to varying conditions, adding a dimension of responsiveness and functionality to the traditional 3D printing process. Hydrogel materials have been widely used in 4D printing due to their stimulus response. When subjected to specific external stimuli such as temperature, pH, ion concentration, electric field, and magnetic field, their physical or chemical properties can be changed in a controlled manner. Li et al. used an MXene solution to further transform an MXene hydrogel with a realistic shape after 3D printing and prepared various hydrogel pattern marks [62]. The development of 4D printing technology, which is now in the exploratory stage, is essentially a continuation of the advancement of 3D printing technology. Currently, 4D printing technology has demonstrated significant promise in various fields such as soft robotics, aerospace, healthcare, and so on. The integration of 4D printing with other emerging technologies holds the potential to accelerate the rapid development and expansion of this innovative technology.

#### 3.1.6. Others

Other than the above manufacturing technology, there are laser-induced forward transfer (LIFT), continuous liquid interface production (CLIP), and so on [63,64], as shown in Figure 3g,h. LIFT uses a pulsed laser beam as the driving force to project the preform liquid onto the receiving substrate. The laser generates a two-dimensional pattern under computer control, and the preform liquid is crosslinked and deposited. CLIP uses especially precise control technology so there are parts that need to be cured and parts that do not need to be cured, and are blocked by oxygen, reducing the staircase effect and obtaining isotropic mechanical properties. As the printing platform moves, the object is raised from the preform. Huang et al. used CLIP technology to print SA/PAM hydrogels with MXene nanosheets, and the resulting hydrogels showed strong structural stability and surface adhesion [65]. To further the creation of hydrogel robots, these technologies use very precise mechanical control systems and computer-assisted systems.

### 3.2. Shape-Morphing Design and the Propulsion of the Soft Smart Microrobots

Inspired by nature, a series of biomimic hydrogel microrobots have been prepared using different stimuli-responsiveness and structural designs. The ability to undergo shape changes allows soft smart microrobots to exhibit a wide range of locomotion modes, including crawling, swimming, rolling, and even flying. By leveraging shape-morphing capabilities, these microrobots can mimic and surpass the locomotion strategies found in nature, enabling them to navigate diverse terrains and perform complex tasks with enhanced swiftness and versatility.

**Figure 4:**
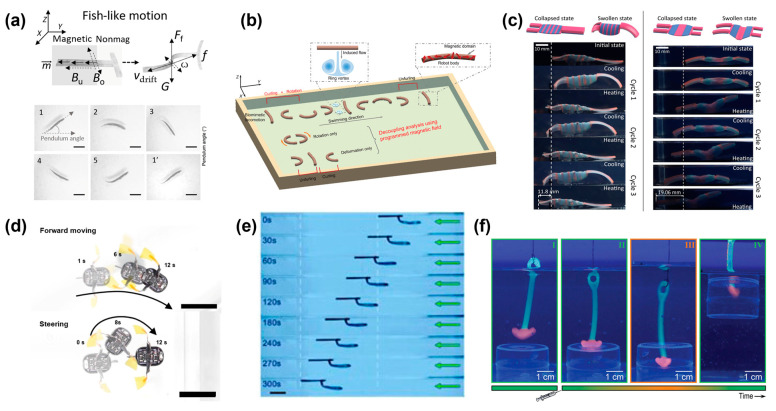
(**a**) Schematic illustration and posture of the magnetic actuation of fish-like locomotion under an oscillating field (B_o_) and a uniform field (B_u_). 1–5 and 1’ represent posture of MMR at different time points. Scale bars, 1 mm. Reproduced with permission [55]. Copyright 2022. The Authors, some rights reserved, exclusive licensee American Association for the Advancement of Science. (**b**) Schematics of the LarvaBot with a variety of reprogrammable coupled gaits, including the biomimetic swimming gait and the decoupling of basic motion. Reproduced with permission [72]. Copyright 2022, Wiley-VCH. (**c**) Illustration and experimental images of a robot at the end of each heating and cooling half-cycle over three consecutive cycles. Reproduced with permission [73]. Copyright 2022. The Authors, some rights reserved, exclusive licensee American Association for the Advancement of Science. (**d**) Photographic images of forward moving and steering soft aquabots with a sculling motion. Scale bar, 3 cm. Reproduced with permission [19]. Copyright 2022. The Authors, some rights reserved, exclusive licensee American Association for the Advancement of Science. (**e**) Sequential snapshots of a swimmer while shining constant light. Input power was 450 mW. Reproduced with permission [75]. Copyright 2019, The Authors, some rights reserved, exclusive licensee American Association for the Advancement of Science. (**f**) Harpoon device. From I to IV: harpoon being lowered over opening, harpoon head resting on opening and unable to spear object, once fueled, head contracts and passes through opening, harpoon being lifted and taking object with it after re-expansion of head. Carried out in 5.0 mm MOPS with 5.0 mm TBA fuel. Reproduced with permission [77]. Copyright 2023, Wiley-VCH.

Magnetic responsiveness is widely used in the current hydrogel-based microrobot. In the process of hydrogel preparation, magnetic nanoparticles are added to the hydrogel; then, the nanoparticles are linearly arranged into axes or uniform distribution by magnetic field manipulation. After the nanoparticles align into the desired design, the hydrogel robot can deform and move under an oscillating magnetic field. This is shown in Zheng’s work. He modularly prepared a T-shaped magnetically responsive hydrogel robot which completed fish-like and caterpillar-like motions under the control of a corresponding magnetic field (Figure 4a) [55]. In addition, a millimeter-scale hydrogel soft robot (larval robot) was fabricated by simulating the natural swimming gait of mosquito larvae. Researchers have conducted decoupling studies on the coupling effects of the curl and rotation of larval robots under magnetic fields [72]. The results showed that their movement ability was significantly correlated with the rotation amplitude and the synchronization of curl and rotation. The larval robot achieved fast movement and upstream movement in the medium Reynolds number range, shown in Figure 4b.

Temperature is a condition that is easy to manipulate in the natural environment. Hydrogel robots produce swelling-induced volume changes under external temperature changes, which has attracted many researchers to utilize this feature to develop the functions of hydrogel robots. PNIPAM is a commonly used temperature-sensitive hydrogel material and researchers have demonstrated the motion mechanism of a one-way crawling gel [73]. The hydrogel robot can perform the same one-way motion on a smooth substrate through multiple thermal cycles like a reptile, thereby realizing the motion of the hydrogel robot that we can see in Figure 4c. The robot is prepared in a double-layer hydrogel configuration using PNIPAM and PAAM. It is an elegant method that uses a double-layer structure to build the desired structure by controlling the strain distribution between two layers of materials.

Applying an electric field is also a very effective method to stimulate the deformation of the hydrogel robot to propel and perform tasks. Researchers have endowed a wrinkled nanofilm electrode on the surface of a hydrogel robot. Capillary-assisted assembly with metal nanoparticles and deswelling-induced wrinkled structures exhibited high conductivity and excellent mechanical deformation [19]. A structure similar to a flying insect was designed, and a potential was applied to the wrinkled nanofilm electrode sandwiched between the hydrogel to achieve controlled expansion, so that the wings on both sides were scratched, and then the hydrogel robot was pushed forward. The flying insect robot is displayed in Figure 4d. A flexible robot in the form of a bat was created by Shin et al. using two distinct hydrogel pattern layers. Between the two hydrogel layers, Au electrodes were placed to improve their conductivity, allowing for local electrical stimulation of the hydrogel robot and control of its jumping behavior [74].

As we discussed in the previous section, light-responsive hydrogels will undergo volume changes due to light intensity, which has inspired much related research on bionic hydrogel robots. Zhao and colleagues developed a flexible actuator driven by rapid and significant volume changes in photo-responsive hydrogels [75]. This actuator achieved high-speed and controlled phototaxis due to its asymmetric structure (Figure 4e). On top of this, researchers have developed a hydrogel actuator that can respond to changes in light intensity, allowing for easy control of the mode conversion between uniform and repeated swimming under continuous light, as well as the ability to change direction as required [76].

Fusi and his colleagues designed a pH-responsive hydrogel robot similar to an anchor [77]. By altering the pH value, the hydrogel robot undergoes deformation, effectively securing the target object and enabling its successful grasping and manipulation, as shown in Figure 4f. Dai et al. made the hydrogel actuator into a valve capable of being opened and closed multiple times in response to varying pH levels [78]. Zhu et al. used the distributed electric field to make a directional and well-structured nanosheet (NS). They successfully achieved the programmable space-time shape transformation and motion of a hydrogel robot under light radiation [79]. In short, a bionic hydrogel robot can be designed to have specific deformation for different tasks. By incorporating smart materials with tailored properties, these microrobots can adapt their shape and size to navigate through narrow spaces or interact with objects in their surroundings. The combination of shape-morphing design and propulsion mechanisms in soft smart microrobots has paved the way for numerous applications in biomedical engineering, including targeted drug delivery, invasive surgeries, and microsurgery.

## 4. Biomedical Applications of Deformable Microrobots

Hydrogel-based robots exhibit excellent biocompatibility while the ability of hydrogel robots to deform the shape in response to stimuli makes them versatile for use in soft intelligent robotics; thus, ongoing research in miniaturization offers significant practical potential for biomedical applications. This chapter provides an overview of various biological applications involving deformable hydrogel microrobots.

### 4.1. Drug Delivery

**Figure 5:**
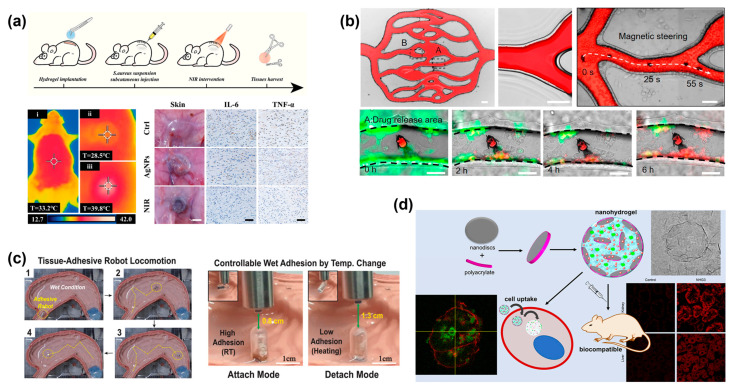
(**a**) Anti-infective therapy of the MXene-based hydrogel system in deep wounds. i–iii represent thermal images of AgNPs-loaded MXene-based hydrogel applied to the rat subcutaneous before and after NIR irradiation. Reproduced with permission [82]. Copyright 2022, Wiley-VCH. (**b**) Localized HeLa cell treatment in a complex network using a magnetic SMMF. A and B: Artificial networks generated by photolithography and PDMS molding. Reproduced with permission [15]. Copyright 2021, American Chemical Society. (**c**) 1–4: In vitro test showing the sophisticated locomotion of the OHA robot to find the target tissue in the body environment, and the tissue adhesion test at different temperatures. Reproduced with permission [83]. Copyright 2022, Wiley-VCH. (**d**) A new nanohydrogel drug delivery platform. Reproduced with permission [85]. Copyright 2018, American Chemical Society.

Hydrogels’ swelling properties offer a favorable scaffold for controlled drug release, leading to the development of diverse hydrogel microrobots for drug delivery. For example, in the study of active motion systems in porous media, researchers developed a soft hydrogel track with remote magnetic response mobility in a limited space [80]. The robots were able to realize reversible contraction and elongation of their bodies to complete peristalsis by repeatedly opening and closing alternating magnetic fields [81]. Yang et al. [82] presented an intelligent hydrogel system that incorporates MXene. This system demonstrated controllable drug delivery capabilities in response to light and magnetic stimuli. Importantly, the hydrogel system proved effective in promoting the healing of deep chronic infected wounds, as demonstrated in Figure 5a. Furthermore, Chen et al. [15] developed a pH-responsive deformable microfish (SMMF) that could encapsulate a drug (doxorubicin) by closing its mouth in phosphate-buffered saline (pH = 7.4) and opening its mouth in a weakly acidic solution (pH < 7). The approach was demonstrated in a scaled-up artificial blood vessel model, showcasing its potential for cancer cell therapy in target areas (as depicted in Figure 5b).

Inspired by the octopus sucker, Li and his colleagues proposed a hydrogel robot that mimics the structure of the octopus sucker and achieved repeatable tissue adhesion and detachment under magnetic control and temperature responsiveness [83]. By incorporating PNIPAM hydrogel spheres into the PEGDA hydrogel wall, a dome-like protrusion structure was formed. The volume change in the PNIPAM hydrogel, triggered by temperature changes, altered the pressure within the structure, enabling controlled adsorption and detachment (as shown in Figure 5c). This paves the way for wireless flexible microrobots to be used for minimally invasive medical interventions, such as drug delivery, in the future. In biological applications, Li et al. developed a thermosensitive hydrogel using a multifunctional crosslinking agent. It could deliver activin B and assess its therapeutic effect in Parkinson’s disease [84]. According to the experimental results, it demonstrated a sustained release of the drug within mice for a period of 35 days, highlighting the potential use in drug delivery and the ability to enhance the therapeutic effect.

Similarly, Becher and his colleagues developed a multifunctional nanohydrogel drug delivery platform based on laponite nanosheets, polyacrylates, and sodium phosphate salts which can encapsulate several cancer drugs simultaneously (Figure 5d). Studies have shown the ability of the hydrogel platform to deliver antitumor drugs to cancer cells while exhibiting biocompatibility without accumulation in vital organs [85]. In addition, a bifunctional isoG-based supramolecular hydrogel with local delivery and anticancer action has been created by Liu and colleagues [86]. The hydrogel demonstrated great pH responsiveness, excellent sustained release capability, and excellent stability (more than a year). The hydrogel significantly inhibited tumor growth (about 60% tumor growth inhibition rate) and improved overall survival rate, especially in the preclinical patient-derived xenograft (PDX) model of oral squamous cell carcinoma (OSCC), according to both in vitro and in vivo experiments. These results emphasize the value of stimuli-responsive hydrogel-based robots as effective drug delivery systems, capable of delivering therapeutic agents to target sites while minimizing off-target effects which opens up new possibilities for personalized medicine and improved treatment strategies in cancer therapy. Mendez et al. introduced a novel hybrid hydrogel actuator (HHA) [87]. The actuator can be securely adhered to tissue through a flexible, drug-permeable adhesive that can withstand the device’s actuation. The controlled release of the charged drug with an adjustable mechanical response was initiated from the alginate/acrylamide hydrogel layer through precise time control. This advancement enhanced the current state of spatial delivery by the hydrogel robot.

### 4.2. Stem Cell Therapy

Cell-based therapy has obtained considerable attention in recent years that involves the injection, grafting, or implantation of cells into the patient [88], aiming to replace [89], repair [90], or enhance the biological function of damaged tissues or systems [91]. Hydrogel-based microrobots have emerged as a valuable tool to accomplish cell delivery, offering cell protection during transportation, maximizing cell viability, and facilitating tissue regeneration in targeted areas [92].

**Figure 6:**
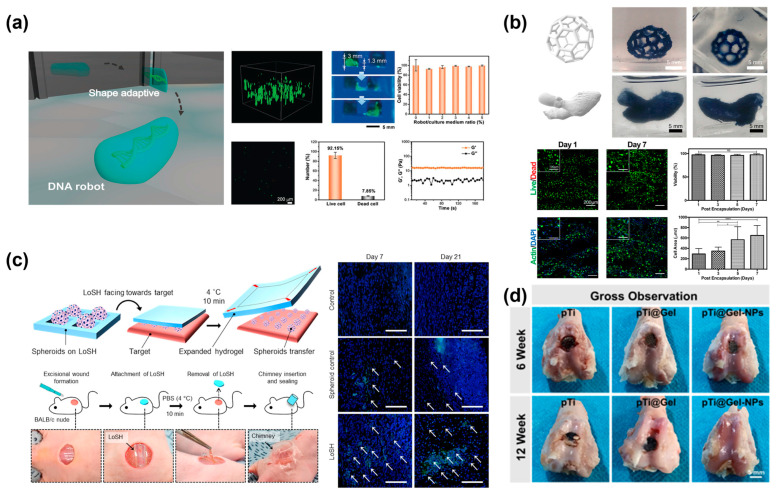
(**a**) DNA robot as a vehicle for cell delivery. Reproduced with permission [23]. Copyright 2020, Wiley-VCH. (**b**) Three-dimensional bioprinting of complex structures using the synthesized GelMA/PEDOT: PSS bioink. Reproduced with permission [93]. Copyright 2019, American Chemical Society. (**c**) Delivery of spheroids to the skin wound model using LoSH. White arrow: fluorescence signals from transplanted cells within sectioned tissue at 7 and 21 days after spheroid transfer (scale bar: 100 μm). Reproduced with permission [94]. Copyright 2019, ELSEVIER. (**d**) Prosthetic interface osseointegration by hydrogel-mediated stem cell therapy. (Data plotted as mean ± SD, * = *p* < 0.05; ** = *p* < 0.01; **** = *p* < 0.0001) Reproduced with permission [95]. Copyright 2022, Springer Nature.

A magnetically controlled ultra-soft and super-elastic flexible robot has been developed. It has shape adaptability and can perform magnetic drive motion in a confined space [23]. The hydrogel robot completes a series of complex magnetic drive motions by altering and restoring its own shape. With its three-dimensional porous network structure and good biocompatibility, these hydrogel robots serve as carriers for cell transportation in restricted spaces, ensuring high cell survival rates exceeding 92%, as demonstrated in Figure 6a. In addition, researchers have also explored a conductive hydrogel composed of gelatin meth acryloyl (GelMA) and poly (3,4-ethylenedioxythiophene):poly(styrene sulfonate) (PEDOT:PSS), exhibiting high cell compatibility and diffusion and biodegradation without a significant inflammatory response, as illustrated in Figure 6b [93]. Furthermore, inspired by lotus seedpods, Kim et al. developed hydrogels with micropores resembling lotus pores for stem cell culture and transportation [94]. At 37 °C, the hydrogel contracted, enabling the loading and transportation of stem cells, while at 4 °C, the hydrogel swelled and led to the separation of stem cells. The results showed that the stem cells were successfully transferred to the target substrate with high transfer efficiency (93.78 ± 2.30%). In mouse experiments, significant increases in blood vessel formation were observed in full-thickness mouse skin wounds with chimney models on the 21st day (Figure 6c).

Furthermore, various modified hydrogel structures have also been developed. Zhao et al. developed a nanozyme-enhanced hydrogel by incorporating metal–organic framework (MOF)-derived nanozymes. This system effectively protected implanted bone marrow-derived mesenchymal stem cells (BMSCs) from reactive oxygen species (ROS) and hypoxia-mediated death (Figure 6d) [95]. These advancements highlight the diverse applications of hydrogel robots in stem cell therapy and regenerative medicine, offering promising solutions for improving therapeutic outcomes.

### 4.3. Cargo Manipulation

Hydrogel microrobots can manipulate bio-samples through their cargo-loading capacity and with their precise movement and localization, they can minimize the impact on healthy tissues or organs, offering a promising approach for targeted sampling or microsurgery in medical diagnosis and disease treatment [78,96,97].

**Figure 7:**
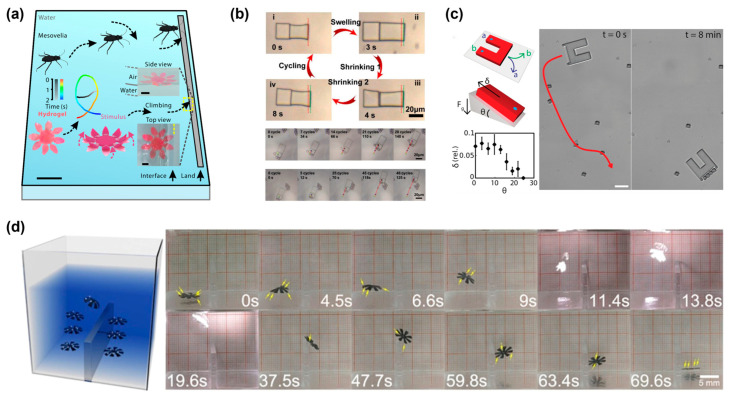
(**a**) All kinds of locomotion of hydrogel water striders and common water striders, including freewill moving on the water surface and menisci climbing. Reproduced with permission [98]. Copyright 2021. The Authors, some rights reserved, exclusive licensee American Association for the Advancement of Science. (**b**) A microparticle carrier. The micro-worm could carry silica microspheres as cargoes and carrying the cargoes through other obstacle microspheres. i–iv: the optical images of the crawling motion of a hydrogel micro-worm in single cycle. Reproduced with permission [100]. Copyright 2020, ELSEVIER. (**c**) The steerable crawler navigated and manipulated 25 μm hydrogel cubes. (**a**,**b**): “U”-shaped crawler is steered by aiming the laser beam at spot (**a**) or (**b**) to turn right and left, respectively. The red arrow indicates the crawler trajectory (the scale bar corresponds to 100 μm). Reproduced with permission [101]. Copyright 2021, Mary Ann Liebert, Inc. (**d**) Transportation of tiny objects by the JMSR. Reproduced with permission [102]. Copyright 2021, American Chemical Society.

One notable development is the self-propelled hydrogel robot capable of navigating through complex environments. Inspired by the bionic water strider, this hydrogel robot can cross obstacles and reach target areas on polluted water surfaces by responding to humidity stimulation (as depicted in Figure 7a) [98]. Zheng et al. designed a programmable deformable hydrogel robot with a petal-shaped structure. This robot utilizes a magnetic field for transportation and pH responsiveness to expand or contract its shape, enabling release and sampling functions in in vitro environments and gastrointestinal tract experiments [99]. Furthermore, a worm-like hydrogel microrobot has been developed that capable of loading silica microspheres and bypassing barrier microspheres under humidity stimulation, demonstrating the potential and versatility of hydrogel microrobots in cargo delivery (Figure 7b) [100]. Hydrogel microrobots for tracked vehicles are designed to utilize light-induced friction hysteresis for control. This feature enables uphill and downhill movement while simultaneously transporting multiple hydrogel cubes without a loss of propulsion, as shown in Figure 7c [101]. Yin et al. introduced jellyfish-like flexible robots to transport microparticles, such as polystyrene spheres. By manipulating the position of the light source, the hydrogel microrobot can bypass obstacles and return under the influence of gravity, illustrated in Figure 7d [102]. All the research works show the potential of hydrogel microrobots in achieving targeted movement, precise delivery, and the controlled release of therapeutic agents. Although the use for bio-sampling or microsurgery is still in the state of proof of concept, the grasping and transporting function can someday be applied in real-world applications.

### 4.4. Minimally Invasive Surgery

Minimally invasive surgery offers several advantages over standard open surgery; notably, leaving fewer and less conspicuous wounds or scars post-procedure. This approach contributes to a faster recovery for patients and can also alleviate some of their postoperative pain. Hydrogels are gaining increasing attention in the field of minimally invasive surgery due to their structural similarities to the extracellular matrix, ease of processing, and potential applications in enhancing surgical techniques, thereby further advancing patient care [103].

Based on two extracellular matrix derivatives, Zhou and his coworkers presented a hydrogel composite material (gelatin and chondroitin sulfate) [104]. This hydrogel was specifically designed for surgical applications, such as sealing or reconnecting torn tissues. It possesses several valuable attributes, including temperature responsiveness, reversible adhesion, and biocompatibility. Due to hydrogen bonding interactions, hydrogel binders have robust tissue adherence at physiological temperatures. At lower temperatures, these interactions weaken, making it easier to detach the hydrogel from tissues. This unique property makes the hydrogel a promising candidate for use as a laparoscopic sealant in minimally invasive surgery. The concept’s validity was demonstrated through experiments on a rat liver injury model, suggesting the potential practicality and effectiveness of this hydrogel as a laparoscopic sealant in surgical procedures. Similarly, Salzlechner et al. developed a hydrogel based on hyaluronic acid (HA) which can be used under aqueous conditions. It exhibits the ability to rapidly gel when exposed to standard surgical lights. Furthermore, it can adhere to tissues and transport cells and potential drugs; it is also suitable for minimally invasive surgery [105], offering a versatile and adaptable material for various surgical procedures. It was demonstrated that adding methacrylate (MA) and 3,4-dihydroxyphenylalanine (Dopa) groups to the hyaluronic acid hydrogel could be a promising approach to facilitate tissue healing in the context of maxillofacial surgeries performed with minimally invasive techniques. Other applications of hydrogel have also been studied in minimally invasive surgery. Sun and his colleagues reported an enzyme-crosslinked hydrogel as a nanoporous hemostatic material that combines a transglutaminase reaction and Schiff base reaction for subcutaneous injection [106]. In pig and rat arterial vascular model experiments, the results showed that the hydrogel coagulation time was about 10 s, and it could effectively stop bleeding without using hemostatic forceps at both ends of the vascular injury. It was proven that the hydrogel acts as an anti-adhesion barrier. Thus, the use of hydrogel is a very significant development for minimally invasive surgery, such as hemostasis, stent, and tissue adhesion to deliver drugs.

### 4.5. Others

Hydrogel microrobots have diverse applications, including carrying medical contrast agents to target positions to provide assistance for MRI and CT medical imaging. Chen and his colleagues demonstrated a new type of medical imaging hydrogel with pH and thermal responsiveness [107]. The effectiveness of medical imaging was confirmed in a mouse model by loading Prussian blue nanoparticles. This approach opens up possibilities for improving the accuracy and specificity of medical imaging. Hydrogels can also be used as wound dressings due to their stretchability. Wu et al. designed a wound dressing for oral wound repair. In vitro experiments have verified that this hydrogel wound dressing can provide lasting protection for intraoral wounds, effectively resisting complex stimuli [108]. Jiang reported a printed double-layer protein hydrogel patch for the treatment of heart failure, showing good results for hemostasis, reduction in fibrosis, and recovery of cardiac function in mice with two types of myocardial injury [109]. Han distributed nano-sized PDA particles into a PVA-PVP hydrogel which increased the hydrogel’s self-adhesion and conductivity and enabled the acquisition of a reliable and stable EEG signal, confirming the hydrogel’s promising applications in biosensors [110].

These examples illustrate the versatility of hydrogel microrobots in various biomedical applications. Hydrogels can serve as carriers to facilitate the targeted delivery of medicine, contribute to wound healing and tissue repair, provide imaging enhancements, and enable biosensing capabilities. The unique properties of hydrogels, such as their biocompatibility, responsiveness to stimuli, and customizable drug release profiles, make them valuable tools in advancing medical diagnostics and treatments.

## 5. Outlook

Compared with other materials, hydrogels have excellent biocompatibility, which provides a new direction for the development of biomaterials, attracts people‘s interest, and promotes the transformation from research to practical application. This paper mainly introduces deformable hydrogel microrobots combined with stimuli responsiveness, such as magnetism, light, electricity, and temperature, and introduces their applications, most notably, in biological applications, including drug delivery, stem cell therapy, cargo handling, and minimally invasive surgery. For example, in minimally invasive surgery, hydrogels can not only serve as drug delivery systems but also as hemostasis and stents, which greatly expands the application prospects of hydrogels. The unique advantages of hydrogel robots, their combination of deformability and biological intelligence, will have a significant impact on the new generation of hydrogel robots.

Although hydrogel robots have made great progress, they also have shortcomings. There are some problems in the design and driving of hydrogel robots, and there are major challenges in achieving biological applications in vivo. From the design point of view, the grasping, deformation degree, and reaction time of the proposed hydrogel robots are still limited, and they are difficult to control accurately. At the same time, how to continuously release drugs/cells in the same location must also be considered when designing hydrogel robots, though the design of the octopus sucker can improve the adsorption on the surface of the target area. At present, the induction of magnetism, humidity, light, and electricity have been widely used by researchers, but the lack of sensitivity and accurate imaging in vivo are still challenges. In in vitro experiments, hydrogel robots can achieve targeted delivery. However, when entering the real internal environment, the factors affecting the hydrogel robot are more complex, and the trajectory of the hydrogel robot cannot be observed in real time.

These are factors restricting the development of hydrogel robots, but through the rational design of hydrogel robot materials and structures, the application prospect of hydrogel robots is very broad. By looking for bionic inspiration in nature, soft and hard hybrid bionic hydrogel robots have been developed for controllable grasping, tumor cell detection, and the continuous release of drugs/cells. Additionally, the hydrogel robot has been combined with a medical contrast agent to observe the imaging of the hydrogel robot in the body through nuclear magnetic resonance technology. Finally, the responsiveness of hydrogel materials has different stimulation conditions; with the innovative development of new design and synthesis strategies, it is expected that a hydrogel robot can release different drugs/cells on time. In addition, with advances in technology, hydrogel robots will better match the patient’s physical condition, revolutionizing the biomedical industry.

## Figures and Tables

**Table 1:** Various stimuli-responsive hydrogel systems for biomedical applications.

Stimulus	Hydrogel	Preparation	Key Feature	Application	References
Temperature	PNIPAm-SA	Sacrificial template method	Shape conversion and color responsiveness	Sensing, communication	[11]
	PNIPAM/PCLDMA/BACy	Polymerization	Thermoresponsive and biodegradable	Drug delivery	[12]
	PPS-b-PDMA-b-PNIPAM	RAFT polymerization	ROS-triggered hydrogel degradation and drug release	Drug delivery	[13]
	NIPAM-AAc and Au@SiO_2_	Polymer encapsulated	NIR light-triggered drug release and thermochem therapy	Drug delivery	[14]
pH	PNIPAm-AAc	4D Printing	Wireless motion control, designable deformation, and encapsulation/release of topical drug (DOX)	Drug delivery	[15]
	PAAm	Thermal polymerization and template	Shape-memory properties	Sensing and drug delivery	[16]
	p(HEMA-co-AA)	Photomask	Reversible structural transformation, in situ encapsulation, and release of microparticles	Drug delivery	[17]
	PAM/Ag	Polymerization	Self-healing performance and anisotropy structure	Tissue engineering	[18]
Electricity	PAA-PAAm	UV-polymerization and capillary-assisted in situ NP assembly	High electrical conductivity, mechanical flexibility, and works at low voltage	Cargo manipulation	[19]
	rGO/poly (AMPS-co-AAm)	Thermal polymerization	Rapid, significant, and reversible electro-response	Cargo manipulation	[20]
	VBS/HEMA/AAm	UV-polymerization and template	2D–3D free conversion	Cargo manipulation	[21]
Magnetics	NIPAm-PEGDA/Fe_3_O_4_	Photomask	Self-folding, patterning, and programmable	Drug delivery	[22]
	PAAm/DNA/Fe_3_O_4_	Permanent crosslinking and dynamic crosslinking	Shape adaptability and accurate navigation locomotion	Drug delivery	[23]
	DMAAm/Laponite/Fe_3_O_4_	In situ free-radical polymerization	High mechanical and excellent biocompatibility	Cargo manipulation	[24]
Light	PPY-GO	Polymerization and template	Smart and programmable	Cargo manipulation	[25]
	AG-PCAD	Polymerization and template	Convert humidity gradients into electrical power in low-power generators and transport cargo close to 20-fold their own weight	Cargo manipulation	[26]
Others	NFC/LiCl	Polymerization	Untethered, self-powered, and humidity responsive	Sensing	[27]
	PEO/PI	Directional electrospinning	Crawling driven by spatial humidity gradient	Sensing and cargo manipulation	[28]
	Poly (sodium acrylate)	Ionoprinting	Responsiveness of organic solvents	Drug delivery and cargo manipulation	[29]
	PAAm-alginate	Template	Hydraulic-driven and fatigue-resistant	Optical camouflage, ultrasonography, and drug delivery	[30]

**Table 2:** Crosslinking mechanisms of hydrogel.

Type	Crosslinking Mechanism	Crosslinking Method	References
Physical crosslinking	Thermally-induced entanglement of polymer chains	Through the interaction between molecules, the crosslinked structure is formed in the hydrogel.	[31,32]
	Molecular self-assembly		[33,34]
	Ionic gelation		[35]
	Electrostatic interaction		[36,37]
Chemical crosslinking	Free radical polymerization	Using chemical reactions and multi-functional monomers, the monomer compound is polymerized into a polymer to form a hydrogel.	[38]
	Photopolymerization	Under the action of specific light sources and photo initiators, hydrogels are formed by covalent bond crosslinking.	[39]
	Radiation polymerization	The water molecules in the polymer solution are irradiated by high-energy rays to produce a variety of active groups that can react with the polymer to generate polymer free radicals, and the polymer-free radicals are then crosslinked to form a network structure polymer.	[40]

## Data Availability

Data sharing not applicable.

## References

[1] Li J., Li X., Luo T., Wang R., Liu C., Chen S., Li D., Yue J., Cheng S.-H., Sun D. (2018). Development of a magnetic microrobot for carrying and delivering targeted cells. Sci. Robot..

[2] Piantanida E., Alonci G., Bertucci A., De Cola L. (2019). Design of Nanocomposite Injectable Hydrogels for Minimally Invasive Surgery. Acc. Chem. Res..

[3] Li F., Lyu D., Liu S., Guo W. (2019). DNA Hydrogels and Microgels for Biosensing and Biomedical Applications. Adv. Mater..

[4] Yoon D., Park S., Park S. (2023). Smart hydrogel structure for microbiome sampling in gastrointestinal tract. Sens. Actuators B Chem..

[5] Zhang Y.S., Khademhosseini A. (2017). Advances in engineering hydrogels. Science.

[6] Zhao Z., Wang Z., Li G., Cai Z., Wu J., Wang L., Deng L., Cai M., Cui W. (2021). Injectable Microfluidic Hydrogel Microspheres for Cell and Drug Delivery. Adv. Funct. Mater..

[7] Jiang Y., Wang J., Zhang H., Chen G., Zhao Y. (2022). Bio-inspired natural platelet hydrogels for wound healing. Sci. Bull..

[8] Liu H., Li M., Liu S., Jia P., Guo X., Feng S., Lu T.J., Yang H., Li F., Xu F. (2020). Spatially modulated stiffness on hydrogels for soft and stretchable integrated electronics. Mater. Horiz..

[9] Jiao D., Zhu Q.L., Li C.Y., Zheng Q., Wu Z.L. (2022). Programmable Morphing Hydrogels for Soft Actuators and Robots: From Structure Designs to Active Functions. Acc. Chem. Res..

[10] Ganguly S., Margel S. (2020). Review: Remotely controlled magneto-regulation of therapeutics from magnetoelastic gel matrices. Biotechnol. Adv..

[11] Wang Y., Zhang Z., Chen H., Zhang H., Zhang H., Zhao Y. (2022). Bio-inspired shape-memory structural color hydrogel film. Sci. Bull..

[12] Gan J., Guan X., Zheng J., Guo H., Wu K., Liang L., Lu M. (2016). Biodegradable, thermoresponsive PNIPAM-based hydrogel scaffolds for the sustained release of levofloxacin. RSC Adv..

[13] Gupta M.K., Martin J.R., Werfel T.A., Shen T., Page J.M., Duvall C.L. (2014). Cell Protective, ABC Triblock Polymer-Based Thermoresponsive Hydrogels with ROS-Triggered Degradation and Drug Release. J. Am. Chem. Soc..

[14] Zhang Z., Wang J., Nie X., Wen T., Ji Y., Wu X., Zhao Y., Chen C. (2014). Near Infrared Laser-Induced Targeted Cancer Therapy Using Thermoresponsive Polymer Encapsulated Gold Nanorods. J. Am. Chem. Soc..

[15] Xin C., Jin D., Hu Y., Yang L., Li R., Wang L., Ren Z., Wang D., Ji S., Hu K. (2021). Environmentally Adaptive Shape-Morphing Microrobots for Localized Cancer Cell Treatment. ACS Nano.

[16] Guo W., Lu C.-H., Orbach R., Wang F., Qi X.-J., Cecconello A., Seliktar D., Willner I. (2014). pH-Stimulated DNA Hydrogels Exhibiting Shape-Memory Properties. Adv. Mater..

[17] Shim T.S., Kim S.-H., Heo C.-J., Jeon H.C., Yang S.-M. (2011). Controlled Origami Folding of Hydrogel Bilayers with Sustained Reversibility for Robust Microcarriers. Angew. Chem. Int. Ed..

[18] Qin H., Zhang T., Li N., Cong H.-P., Yu S.-H. (2019). Anisotropic and self-healing hydrogels with multi-responsive actuating capability. Nat. Commun..

[19] Ko J., Kim C., Kim D., Song Y., Lee S., Yeom B., Huh J., Han S., Kang D., Koh J.-S. (2022). High-performance electrified hydrogel actuators based on wrinkled nanomembrane electrodes for untethered insect-scale soft aquabots. Sci. Robot..

[20] Yang C., Liu Z., Chen C., Shi K., Zhang L., Ju X.-J., Wang W., Xie R., Chu L.-Y. (2017). Reduced Graphene Oxide-Containing Smart Hydrogels with Excellent Electro-Response and Mechanical Properties for Soft Actuators. ACS Appl. Mater. Interfaces.

[21] Choi M.-Y., Shin Y., Lee H.S., Kim S.Y., Na J.-H. (2020). Multipolar spatial electric field modulation for freeform electroactive hydrogel actuation. Sci. Rep..

[22] Huang H.-W., Sakar M.S., Petruska A.J., Pané S., Nelson B.J. (2016). Soft micromachines with programmable motility and morphology. Nat. Commun..

[23] Tang J., Yao C., Gu Z., Jung S., Luo D., Yang D. (2020). Super-Soft and Super-Elastic DNA Robot with Magnetically Driven Navigational Locomotion for Cell Delivery in Confined Space. Angew. Chem. Int. Ed..

[24] Lee J.H., Han W.J., Jang H.S., Choi H.J. (2019). Highly Tough, Biocompatible, and Magneto-Responsive Fe_3_O_4_/Laponite/PDMAAm Nanocomposite Hydrogels. Sci. Rep..

[25] Dong Y., Wang J., Guo X., Yang S., Ozen M.O., Chen P., Liu X., Du W., Xiao F., Demirci U. (2019). Multi-stimuli-responsive programmable biomimetic actuator. Nat. Commun..

[26] Zhang L., Liang H., Jacob J., Naumov P. (2015). Photogated humidity-driven motility. Nat. Commun..

[27] Wang Y., Dai M., Wu H., Xu L., Zhang T., Chen W., Wang Z.L., Yang Y. (2021). Moisture induced electricity for self-powered microrobots. Nano Energy.

[28] Shin B., Ha J., Lee M., Park K., Park G.H., Choi T.H., Cho K.-J., Kim H.-Y. (2018). Hygrobot: A self-locomotive ratcheted actuator powered by environmental humidity. Sci. Robot..

[29] Palleau E., Morales D., Dickey M.D., Velev O.D. (2013). Reversible patterning and actuation of hydrogels by electrically assisted ionoprinting. Nat. Commun..

[30] Yuk H., Lin S., Ma C., Takaffoli M., Fang N.X., Zhao X. (2017). Hydraulic hydrogel actuators and robots optically and sonically camouflaged in water. Nat. Commun..

[31] Lutz J.-F., Akdemir Ö., Hoth A. (2006). Point by Point Comparison of Two Thermosensitive Polymers Exhibiting a Similar LCST: Is the Age of Poly(NIPAM) Over?. J. Am. Chem. Soc..

[32] Ward M.A., Georgiou T.K. (2011). Thermoresponsive Polymers for Biomedical Applications. Polymers.

[33] Chen H., Liu Y., Ren B., Zhang Y., Ma J., Xu L., Chen Q., Zheng J. (2017). Super Bulk and Interfacial Toughness of Physically Crosslinked Double-Network Hydrogels. Adv. Funct. Mater..

[34] O’Leary L.E.R., Fallas J.A., Bakota E.L., Kang M.K., Hartgerink J.D. (2011). Multi-hierarchical self-assembly of a collagen mimetic peptide from triple helix to nanofibre and hydrogel. Nat. Chem..

[35] Rowley J.A., Madlambayan G., Mooney D.J. (1999). Alginate hydrogels as synthetic extracellular matrix materials. Biomaterials.

[36] Morton S.W., Herlihy K.P., Shopsowitz K.E., Deng Z.J., Chu K.S., Bowerman C.J., DeSimone J.M., Hammond P.T. (2013). Scalable Manufacture of Built-to-Order Nanomedicine: Spray-Assisted Layer-by-Layer Functionalization of PRINT Nanoparticles. Adv. Mater..

[37] Leijten J., Rouwkema J., Zhang Y.S., Nasajpour A., Dokmeci M.R., Khademhosseini A. (2016). Tissue Engineering: Advancing Tissue Engineering: A Tale of Nano-, Micro-, and Macroscale Integration (Small 16/2016). Small.

[38] DeForest C.A., Polizzotti B.D., Anseth K.S. (2009). Sequential click reactions for synthesizing and patterning three-dimensional cell microenvironments. Nat. Mater..

[39] DeForest C.A., Anseth K.S. (2011). Cytocompatible click-based hydrogels with dynamically tunable properties through orthogonal photoconjugation and photocleavage reactions. Nat. Chem..

[40] Sun J.-Y., Zhao X., Illeperuma W.R.K., Chaudhuri O., Oh K.H., Mooney D.J., Vlassak J.J., Suo Z. (2012). Highly stretchable and tough hydrogels. Nature.

[41] Shi Q., Liu H., Tang D., Li Y., Li X., Xu F. (2019). Bioactuators based on stimulus-responsive hydrogels and their emerging biomedical applications. NPG Asia Mater..

[42] Bai L., Jin Y., Shang X., Jin H., Shi L., Li Y., Zhou Y. (2022). Temperature-triggered smart milk-derived hydrogel with programmable adhesion for versatile skin-attached iontronics. Nano Energy.

[43] Takezawa T., Mori Y., Yoshizato K. (1990). Cell Culture on a Thermo-Responsive Polymer Surface. Nat. Biotechnol..

[44] Guo H., Sanson N., Hourdet D., Marcellan A. (2016). Thermoresponsive Toughening with Crack Bifurcation in Phase-Separated Hydrogels under Isochoric Conditions. Adv. Mater..

[45] Na H., Kang Y.-W., Park C.S., Jung S., Kim H.-Y., Sun J.-Y. (2022). Hydrogel-based strong and fast actuators by electroosmotic turgor pressure. Science.

[46] Haider H., Yang C.H., Zheng W.J., Yang J.H., Wang M.X., Yang S., Zrínyi M., Osada Y., Suo Z., Zhang Q. (2015). Exceptionally tough and notch-insensitive magnetic hydrogels. Soft Matter.

[47] Li L., Scheiger J.M., Levkin P.A. (2019). Design and Applications of Photoresponsive Hydrogels. Adv. Mater..

[48] Zhang F., Fan J., Zhang P., Liu M., Meng J., Jiang L., Wang S. (2017). A monolithic hydro/organo macro copolymer actuator synthesized via interfacial copolymerization. NPG Asia Mater..

[49] Zhu C.N., Li C.Y., Wang H., Hong W., Huang F., Zheng Q., Wu Z.L. (2021). Reconstructable Gradient Structures and Reprogrammable 3D Deformations of Hydrogels with Coumarin Units as the Photolabile Crosslinks. Adv. Mater..

[50] Dai C., Li Z., Li Z., Shi Y., Wang Z., Wan S., Tang J., Zeng Y., Li Z. (2022). Direct-Printing Hydrogel-Based Platform for Humidity-Driven Dynamic Full-Color Printing and Holography. Adv. Funct. Mater..

[51] Nakamoto M., Kitano S., Matsusaki M. (2022). Biomacromolecule-Fueled Transient Volume Phase Transition of a Hydrogel. Angew. Chem. Int. Ed..

[52] Wang X., Chen S., Wu D., Wu Q., Wei Q., He B., Lu Q., Wang Q. (2018). Oxidoreductase-Initiated Radical Polymerizations to Design Hydrogels and Micro/Nanogels: Mechanism, Molding, and Applications. Adv. Mater..

[53] Cui Y., Li D., Gong C., Chang C. (2021). Bioinspired Shape Memory Hydrogel Artificial Muscles Driven by Solvents. ACS Nano.

[54] Li J., Wang M., Cui Z., Liu S., Feng D., Mei G., Zhang R., An B., Qian D., Zhou X. (2022). Dual-responsive jumping actuators by light and humidity. J. Mater. Chem. A.

[55] Zheng Z., Wang H., Demir S.O., Huang Q., Fukuda T., Sitti M. (2022). Programmable aniso-electrodeposited modular hydrogel microrobots. Sci. Adv..

[56] Billiet T., Vandenhaute M., Schelfhout J., Van Vlierberghe S., Dubruel P. (2012). A review of trends and limitations in hydrogel-rapid prototyping for tissue engineering. Biomaterials.

[57] Truby R.L., Lewis J.A. (2016). Printing soft matter in three dimensions. Nature.

[58] Lim K.S., Galarraga J.H., Cui X., Lindberg G.C.J., Burdick J.A., Woodfield T.B.F. (2020). Fundamentals and Applications of Photo-Cross-Linking in Bioprinting. Chem. Rev..

[59] Calvert P. (2001). Inkjet Printing for Materials and Devices. Chem. Mater..

[60] Bedell M.L., Navara A.M., Du Y., Zhang S., Mikos A.G. (2020). Polymeric Systems for Bioprinting. Chem. Rev..

[61] Ozbolat I.T., Hospodiuk M. (2016). Current advances and future perspectives in extrusion-based bioprinting. Biomaterials.

[62] Li K., Zhao J., Zhussupbekova A., Shuck C.E., Hughes L., Dong Y., Barwich S., Vaesen S., Shvets I.V., Möbius M. (2022). 4D printing of MXene hydrogels for high-efficiency pseudocapacitive energy storage. Nat. Commun..

[63] Serra P., Piqué A. (2019). Laser-Induced Forward Transfer: Fundamentals and Applications. Adv. Mater. Technol..

[64] Tumbleston J.R., Shirvanyants D., Ermoshkin N., Janusziewicz R., Johnson A.R., Kelly D., Chen K., Pinschmidt R., Rolland J.P., Ermoshkin A. (2015). Continuous liquid interface production of 3D objects. Science.

[65] Huang B., Zhou Z., Wei L., Song Q., Yu W., Zhou Y., Hu R., Zhang W., Lu C. (2021). Ti_3_C_2_T_x_ MXene as a novel functional photo blocker for stereolithographic 3D printing of multifunctional gels via Continuous Liquid Interface Production. Compos. Part B Eng..

[66] Saadli M., Braunmiller D.L., Mourran A., Crassous J.J. (2023). Thermally and Magnetically Programmable Hydrogel Microactuators. Small.

[67] Zhang Y., Su Y., Zhao Y., Wang Z., Wang C. (2022). Two-Photon 3D Printing in Metal–Organic Framework Single Crystals. Small.

[68] Manapat J.Z., Mangadlao J.D., Tiu B.D.B., Tritchler G.C., Advincula R.C. (2017). High-Strength Stereolithographic 3D Printed Nanocomposites: Graphene Oxide Metastability. ACS Appl. Mater. Interfaces.

[69] Liu Z., Pan W., Wang K., Matia Y., Xu A., Barreiros J.A., Darkes-Burkey C., Giannelis E.P., Mengüç Y., Shepherd R.F. (2021). Acoustophoretic Liquefaction for 3D Printing Ultrahigh-Viscosity Nanoparticle Suspensions. Adv. Mater..

[70] Fu Z., Naghieh S., Xu C., Wang C., Sun W., Chen X. (2021). Printability in extrusion bioprinting. Biofabrication.

[71] Santos M.V., Paula K.T., de Andrade M.B., Gomes E.M., Marques L.F., Ribeiro S.J.L., Mendonça C.R. (2020). Direct Femtosecond Laser Printing of Silk Fibroin Microstructures. ACS Appl. Mater. Interfaces.

[72] Xia N., Jin B., Jin D., Yang Z., Pan C., Wang Q., Ji F., Iacovacci V., Majidi C., Ding Y. (2022). Decoupling and Reprogramming the Wiggling Motion of Midge Larvae Using a Soft Robotic Platform. Adv. Mater..

[73] Pantula A., Datta B., Shi Y., Wang M., Liu J., Deng S., Cowan N.J., Nguyen T.D., Gracias D.H. (2022). Untethered unidirectionally crawling gels driven by asymmetry in contact forces. Sci. Robot..

[74] Shin S.R., Migliori B., Miccoli B., Li Y., Mostafalu P., Seo J., Mandla S., Enrico A., Antona S., Sabarish R. (2018). Electrically Driven Microengineered Bioinspired Soft Robots. Adv. Mater..

[75] Zhao Y., Xuan C., Qian X., Alsaid Y., Hua M., Jin L., He X. (2019). Soft phototactic swimmer based on self-sustained hydrogel oscillator. Sci. Robot..

[76] Li Z., Myung N.V., Yin Y. (2021). Light-powered soft steam engines for self-adaptive oscillation and biomimetic swimming. Sci. Robot..

[77] Fusi G., Del Giudice D., Skarsetz O., Di Stefano S., Walther A. (2023). Autonomous Soft Robots Empowered by Chemical Reaction Networks. Adv. Mater..

[78] Dai L., Ma M., Xu J., Si C., Wang X., Liu Z., Ni Y. (2020). All-Lignin-Based Hydrogel with Fast pH-Stimuli Responsiveness for Mechanical Switching and Actuation. Chem. Mater..

[79] Zhu Q.L., Dai C.F., Wagner D., Daab M., Hong W., Breu J., Zheng Q., Wu Z.L. (2020). Distributed Electric Field Induces Orientations of Nanosheets to Prepare Hydrogels with Elaborate Ordered Structures and Programmed Deformations. Adv. Mater..

[80] Cabanach P., Pena-Francesch A., Sheehan D., Bozuyuk U., Yasa O., Borros S., Sitti M. (2020). Zwitterionic 3D-Printed Non-Immunogenic Stealth Microrobots. Adv. Mater..

[81] Shen T., Font M.G., Jung S., Gabriel M.L., Stoykovich M.P., Vernerey F.J. (2017). Remotely Triggered Locomotion of Hydrogel Mag-bots in Confined Spaces. Sci. Rep..

[82] Yang X., Zhang C., Deng D., Gu Y., Wang H., Zhong Q. (2022). Multiple Stimuli-Responsive MXene-Based Hydrogel as Intelligent Drug Delivery Carriers for Deep Chronic Wound Healing. Small.

[83] Lee Y., Chun S., Son D., Hu X., Schneider M., Sitti M. (2022). A Tissue Adhesion-Controllable and Biocompatible Small-Scale Hydrogel Adhesive Robot. Adv. Mater..

[84] Li J., Darabi M., Gu J., Shi J., Xue J., Huang L., Liu Y., Zhang L., Liu N., Zhong W. (2016). A drug delivery hydrogel system based on activin B for Parkinson’s disease. Biomaterials.

[85] Becher T.B., Mendonça M.C.P., de Farias M.A., Portugal R.V., de Jesus M.B., Ornelas C. (2018). Soft Nanohydrogels Based on Laponite Nanodiscs: A Versatile Drug Delivery Platform for Theranostics and Drug Cocktails. ACS Appl. Mater. Interfaces.

[86] Liu T., Du Y., Yan Y., Song S., Qi J., Xia X., Hu X., Chen Q., Liu J., Zeng X. (2023). pH-responsive dual-functional hydrogel integrating localized delivery and anti-cancer activities for highly effective therapy in PDX of OSCC. Mater. Today.

[87] Mendez K., Whyte W., Freedman B.R., Fan Y., Varela C.E., Singh M., Cintron-Cruz J.C., Rothenbücher S.E., Li J., Mooney D.J. (2023). Mechanoresponsive Drug Release from a Flexible, Tissue-Adherent, Hybrid Hydrogel Actuator. Adv. Mater..

[88] Chen J., Li J., Sun X., Lu H., Liu K., Li Z., Guan J., Song H., Wei W., Ge Y. (2023). Precision Therapy of Recurrent Breast Cancer through Targeting Different Malignant Tumor Cells with a HER2/CD44-Targeted Hydrogel Nanobot. Small.

[89] Fang Y., Guo Y., Ji M., Li B., Guo Y., Zhu J., Zhang T., Xiong Z. (2021). 3D Printing of Cell-Laden Microgel-Based Biphasic Bioink with Heterogeneous Microenvironment for Biomedical Applications. Adv. Funct. Mater..

[90] Ding A., Jeon O., Tang R., Bin Lee Y., Lee S.J., Alsberg E. (2021). Cell-Laden Multiple-Step and Reversible 4D Hydrogel Actuators to Mimic Dynamic Tissue Morphogenesis. Adv. Sci..

[91] Spector M. (2018). Biomedical materials to meet the challenges of the aging epidemic. Biomed. Mater..

[92] Aguado B.A., Mulyasasmita W., Su J., Lampe K.J., Heilshorn S.C., Gillispie G.J., Han A., Uzun-Per M., Fisher J., Mikos A.G. (2012). Improving Viability of Stem Cells During Syringe Needle Flow Through the Design of Hydrogel Cell Carriers. Tissue Eng. Part A.

[93] Spencer A.R., Shirzaei Sani E., Soucy J.R., Corbet C.C., Primbetova A., Koppes R.A., Annabi N. (2019). Bioprinting of a Cell-Laden Conductive Hydrogel Composite. ACS Appl. Mater. Interfaces.

[94] Kim S.-J., Park J., Kim E.M., Choi J.-J., Kim H.-N., Chin I.L., Choi Y.S., Moon S.-H., Shin H. (2019). Lotus seedpod-inspired hydrogels as an all-in-one platform for culture and delivery of stem cell spheroids. Biomaterials.

[95] Zhao Y., Song S., Wang D., Liu H., Zhang J., Li Z., Wang J., Ren X., Zhao Y. (2022). Nanozyme-reinforced hydrogel as a H2O2-driven oxygenerator for enhancing prosthetic interface osseointegration in rheumatoid arthritis therapy. Nat. Commun..

[96] Such G.K., Yan Y., Johnston A.P.R., Gunawan S.T., Caruso F. (2015). Interfacing Materials Science and Biology for Drug Carrier Design. Adv. Mater..

[97] Wang X., Huang H., Liu H., Rehfeldt F., Wang X., Zhang K. (2019). Multi-Responsive Bilayer Hydrogel Actuators with Programmable and Precisely Tunable Motions. Macromol. Chem. Phys..

[98] Zhu H., Xu B., Wang Y., Pan X., Qu Z., Mei Y. (2021). Self-powered locomotion of a hydrogel water strider. Sci. Robot..

[99] Zheng Z., Wang H., Dong L., Shi Q., Li J., Sun T., Huang Q., Fukuda T. (2021). Ionic shape-morphing microrobotic end-effectors for environmentally adaptive targeting, releasing, and sampling. Nat. Commun..

[100] Sun X.-C., Xia H., Xu X.-L., Lv C., Zhao Y. (2020). Ingenious humidity-powered micro-worm with asymmetric biped from single hydrogel. Sens. Actuators B Chem..

[101] Rehor I., Maslen C., Moerman P.G., van Ravensteijn B.G., van Alst R., Groenewold J., Eral H.B., Kegel W.K. (2021). Photoresponsive Hydrogel Microcrawlers Exploit Friction Hysteresis to Crawl by Reciprocal Actuation. Soft Robot..

[102] Yin C., Wei F., Fu S., Zhai Z., Ge Z., Yao L., Jiang M., Liu M. (2021). Visible Light-Driven Jellyfish-like Miniature Swimming Soft Robot. ACS Appl. Mater. Interfaces.

[103] Raucci M.G., D’Amora U., Ronca A., Ambrosio L. (2020). Injectable Functional Biomaterials for Minimally Invasive Surgery. Adv. Healthc. Mater..

[104] Zhou L., Dai C., Fan L., Jiang Y., Liu C., Zhou Z., Guan P., Tian Y., Xing J., Li X. (2021). Injectable Self-Healing Natural Biopolymer-Based Hydrogel Adhesive with Thermoresponsive Reversible Adhesion for Minimally Invasive Surgery. Adv. Funct. Mater..

[105] Salzlechner C., Haghighi T., Huebscher I., Walther A.R., Schell S., Gardner A., Undt G., da Silva R.M.P., Dreiss C.A., Fan K. (2020). Adhesive Hydrogels for Maxillofacial Tissue Regeneration Using Minimally Invasive Procedures. Adv. Healthc. Mater..

[106] Sun D., Wang H., Liu J., Wang X., Guo H., Xue L., Li L., Li J., Zhang B., Xue Y. (2022). An enzyme cross-linked hydrogel as a minimally invasive arterial tissue sealing and anti-adhesion barrier. Nano Today.

[107] Chen Z., Chen R., Zhao C., Quan Z., Zhu H., Wang L., Bu Q., He Y., He H. (2022). A novel medically imageable intelligent cellulose nanofibril-based injectable hydrogel for the chemo-photothermal therapy of tumors. Chem. Eng. J..

[108] Wu J., Pan Z., Zhao Z.Y., Wang M.H., Dong L., Gao H.L., Liu C.Y., Zhou P., Chen L., Shi C.J. (2022). Anti-Swelling, Robust, and Adhesive Extracellular Matrix-Mimicking Hydrogel Used as Intraoral Dressing. Adv. Mater..

[109] Jiang X., Feng T., An B., Ren S., Meng J., Li K., Liu S., Wu H., Zhang H., Zhong C. (2022). A Bi-Layer Hydrogel Cardiac Patch Made of Recombinant Functional Proteins. Adv. Mater..

[110] Han Q., Zhang C., Guo T., Tian Y., Song W., Lei J., Li Q., Wang A., Zhang M., Bai S. (2023). Hydrogel Nanoarchitectonics of a Flexible and Self-Adhesive Electrode for Long-Term Wireless Electroencephalogram Recording and High-Accuracy Sustained Attention Evaluation. Adv. Mater..

